# Phenotype/genotype sequence complementarity and prebiotic replicator coexistence in the metabolically coupled replicator system

**DOI:** 10.1186/s12862-014-0234-8

**Published:** 2014-11-25

**Authors:** Balázs Könnyű, Tamás Czárán

**Affiliations:** Department of Plant Systematics, Ecology and Theoretical Biology, Eötvös Loránd University, Pázmány Péter sétány 1/c, Budapest, 1117 Hungary; MTA-ELTE Theoretical Biology and Evolutionary Ecology Research Group, Eötvös Loránd University, Pázmány Péter sétány 1/c, Budapest, 1117 Hungary

**Keywords:** Prebiotic evolution, Replicator, RNA World, Coexistence, Complementary RNA strains, Phenotype, Genotype, Template replication

## Abstract

**Background:**

RNA or RNA-like polymers are the most likely candidates for having played the lead roles on the stage of the origin of life. RNA is known to feature two of the three essential functions of living entities (metabolism, heredity and membrane): it is capable of unlimited heredity and it has a proven capacity for catalysing very different chemical reactions which may form simple metabolic networks. The Metabolically Coupled Replicator System is a class of simulation models built on these two functions to show that an RNA World scenario for the origin of life is ecologically feasible, provided that it is played on mineral surfaces. The fact that RNA templates and their copies are of complementary base sequences has an obvious dynamical relevance: complementary strains may have very different structures and, consequently, functions – one may specialize for increasing enzymatic activity while the other takes the role of the gene of the enzyme.

**Results:**

Incorporating the functional divergence of template and copy into the Metabolically Coupled Replicator System model framework we show that sequence complementarity 1) does not ruin the coexistence of a set of metabolically cooperating replicators; 2) the replicator system remains resistant to, but also tolerant with its parasites; 3) opens the way to the evolutionary differentiation of phenotype and genotype through a primitive version of phenotype amplification.

**Conclusions:**

The functional asymmetry of complementary RNA strains results in a shift of phenotype/genotype (enzyme/gene) proportions in MCRS, favouring a slight genotype dominance. This asymmetry is expected to reverse due to the evolved trade-off of high “gene” replicability and high catalytic activity of the corresponding “enzyme” in expense of its replicability. This trade-off is the first evolutionary step towards the “division of labour” among enzymes and genes, which has concluded in the extreme form of *phenotype amplification* characteristic of our recent DNA-RNA-protein World.

**Electronic supplementary material:**

The online version of this article (doi:10.1186/s12862-014-0234-8) contains supplementary material, which is available to authorized users.

## Background

**Figure 1:**
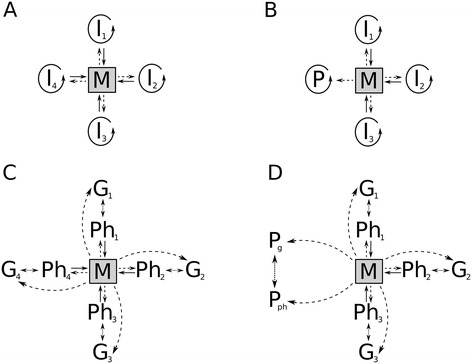
**The scheme of the Metabolically Coupled Replicator System without and with template-directed replication.** *Panel* **A**: The original metabolic replicator system based on [6]. Four autocatalytic metabolic replicators (*I*_*i*_, *i* = 1, .., 4 within the circular arrows). *M* is the metabolic reaction network supported by the metabolic replicators as enzymes (solid lines) and producing monomers for their replication (dashed lines). *Panel* **B**: the same as *Panel* **A** but the system contains a parasitic (*P*) replicator besides metabolic (*I*_*i*_, where *i* = 1..3) ones. Parasites consume monomers produced by the metabolic network but do not contribute to the production of monomers. *Panel* **C**: the Metabolically Coupled Replicator System in which all reactions of a hypothetical metabolic-network (*M*) are catalysed (solid arrows) by the phenotype forms of replicators (*Ph*_*x*_ *,* where *x* = 1, …, 4). Dashed arrows show that replicators consume the end-products of the metabolic-network (monomers). Dotted, double-headed arrows depict template-dependent replication process which produces phenotype from genotype (*G*_*x*_) during a replication event, and *vice versa*. *Panel* **D**: shows parasites in the same system like in *Panel* **C**. *P*_*g*_ and *P*_*ph*_ are the two forms of the parasite.

In a series of simulation studies [1-6] we have shown earlier that a plausible scenario for the prebiotic origins of life can be based on the surface-bound RNA World hypothesis [7-9]. The Metabolically Coupled Replicator System (MCRS) model framework has been developed to demonstrate that a set of different RNA-like macromolecular replicators (ribozymes) cooperating for the production of their own monomers (nucleotides, [10]) can maintain a stable replicator community in which each molecular species (replicator type) catalyses a single reaction of a primitive metabolic reaction network ([1], Figure 1A). The mutualistic interactions among the different catalytic replicator species comprising the MCRS are mediated by their common replication resource – the monomers – produced by the replicators themselves through their close chemical cooperation in processing “nutrient” compounds supplied from the environment. A working metabolism can be maintained only if each metabolically essential species persists, in spite of the inevitable differences in their overall replicabilities which, in a well-mixed medium, would result in the competitive exclusion of all but the fastest-replicating species and thus in the demise of the metabolic network [1]. Charged mineral surfaces might have offered the first escape for cooperating metabolic replicators from the competitive collapse of their communities [11-13], by anchoring the replicators [14,15] and thus preventing their extensive spatial mixing [1,2]. This simple spatial constraint results in negative frequency dependent feedback regulation: slowly replicating (rare) species enjoy the fitness advantage of a high probability of local metabolic complementation to compensate for their inferior replicabilities. The crucial advantage of rarity comes from the local nature of metabolic interactions on the mineral surface: rare metabolic replicator types have a higher chance to find at least one copy of the more common species within the surface-diffusion range of the metabolites than the common ones to have at least one rare type copy nearby (Additional file 1: Figure S1). This simple spatial regulatory mechanism has been shown to maintain robust coexistence in spite of vast differences assumed in the replicabilities of the different replicator species [1,6].

The original MCRS is a toy model neglecting important physical-chemical details of the supposed RNA World scenario [9]. Some of those details and their consequences for the viability of the metabolic replicator system have been considered in specific modifications of the model during the past decade. Such modifications include the presence of parasites – replicators not contributing to the production but taking part in the consumption of the monomers supplied by cooperating species ([1,6], Figure 1B and D) – which were shown to persist in the system but generically unable to drive it extinct. Parasites have been proven capable of evolving functions beneficial for the metabolic system, and thus also for themselves: they can acquire new catalytic activities which may improve metabolic efficiency, or they might evolve a slightly better replicase activity than previously available [3], and thus increase the replication rate of the whole replicator community [5].

In the present study we address the dynamical consequences of the fact that the replication of RNA or any known RNA-like macromolecule yields a copy with a monomer sequence different from that of the template – a property directly following from nucleotide base-pairing rules (A to U, C to G in the case of conventional nucleotide bases). The complementary string of nucleotides in the copy carries the same sequence information as the original (one strain determines the other and *vice versa*), but its 3D structure, and, consequently, its physico-chemical properties – like its enzymatic activity [10] – may be radically different [16]. If the template has an enzymatic activity essential for speeding up a certain reaction step of metabolism, then the copy is almost certainly inactive in that reaction. For the metabolic replicator system as a whole this means that the template is an “enzyme”, but the copy is not; it plays the role of a “gene” instead. It is only the “grand-daughter” of a certain template (i.e., a copy of its copy) that has the metabolic activity of the template. Thus, subsequent generations of the same sequence are competitors of each other, besides their obvious genetic relation. It is easy to see that the MCRS model might be very sensitive to this detail: template directed replication is a dynamical issue requiring closer study.

## Methods

### The MCRS model with phenotype/genotype distinction

The original (single-sequence) toy model framework (MCRS) has been specified in detail in our previous publications [1,3,5,6,17], therefore we confine model description to the essentials here, with emphasis on new assumptions related to complementary sequence replication.

MCRS is implemented as a stochastic cellular automaton (SCA) model on a square lattice representing the mineral surface to which the replicators are anchored. The topology of the lattice is toroidal (opposite sides merged), to avoid edge effects on the dynamics. Each site may be empty or occupied by a single replicator *i* at any point of time. Each replicator type has two forms different with respect to their enzymatic activities and complementary sequence-wise: the *phenotype form* (*enzymatically active*) and the *genotype form* (*enzymatically inactive*, Figure 1C and D). Thus the number of possible different states for a grid site is 2*n* +1, where *n* is the number of replicator types (species), i.e., the *size* of the system.

### Basic assumptions of the surface-bound Metabolically Coupled Replicator System

Each grid site is updated once on average in one generation time using *asynchronous random update*. The first step of the updating algorithm is the random choice of a grid site, the next state of which (at time *t* +1) depends on its own state and those of its neighbours at time *t*. If the focal site is occupied by a replicator, then it becomes empty with a constant probability *p*_*d*_. If it is empty, then it may remain so with probability *p*_*e*_ (Eq. 1), or one of the replicators occupying adjacent sites (from within the *replication neighbourhood* of the focal empty site, Additional file 1: Figure S1) puts a *complementary copy* of itself onto the empty site with probability *p*_*i*_ (Eq. 2).

1 $$ {p}_e=\frac{C_e}{C_e+{\displaystyle \sum_{j=1}^r{C}_j}}, $$

and

2 $$ {p}_i=\frac{C_i}{C_e+{\displaystyle \sum_{j=1}^r{C}_j}}, $$

where *C*_*i*_ and *C*_*j*_ are the “claims” of competing replicators to occupy the empty site and *C*_*e*_ is the “claim” of the empty site to remain empty (in which case replication does not take place at all). *r* is the size of replication neighbourhoods (i.e., the maximum number of replicators possibly competing for an empty site). The size of the replication neighbourhood is a parameter of the model: increasing *r* means that the template may be further away from the empty site on which it claims to place a copy of itself.

The next state of the empty site is determined by a random draw using probabilities *p*_*e*_ and *p*_*i*_. $\left( Note\  that\kern0.5em {p}_e+{\displaystyle \sum_{i=1}^r{p}_i}=1\kern0.5em \right).$

The “claim” of a replicator to put a complementary copy of itself to the focal empty site depends on two components: *k*_*i*_ , a replicator specific constant (*replicability*), and the local metabolic (monomer) supply (*M*_*i*_) of replicator *i*:

3 $$ {C}_i={k}_i\cdot {M}_i $$

### Phenotype-genotype difference in catalytic activity

The local monomer supply *M*_*i*_ of replicator *i* depends on the presence of the *enzymatically active* phenotype forms of *all* the metabolic replicator types, each type with at least one copy, within the *metabolic neighbourhood* of replicator *i* (Additional file 1: Figure S1). That is, only those replicators can produce daughter copies of themselves which have all the necessary metabolic enzymes for monomer production at their disposal within a small distance – any one of the enzymes missing from the metabolic neighbourhood of replicator *i* ruins local metabolism and thus excludes the replication of *i*. The formula satisfying these assumptions for the monomer supply *M*_*i*_ of replicator *i* is

4 $$ {M}_j=\sqrt[n]{{\displaystyle \prod_{j=1}^n{x}_j^{Ph}}}, $$

the geometric mean of the copy numbers (*x*_*j*_^*Ph*^) of all the different phenotype-forms *j* within the metabolic neighbourhood of replicator *i*: *h* is the size of the metabolic neighbourhood (i.e., the number of sites it includes). *M*_*i*_ is either zero (if the enzymatically active metabolic replicator set within the metabolic neighbourhood of site *i* is incomplete), or it is greater than or equal to 1. Obviously, *M*_*i*_ = 0 implies *C*_*i*_ = 0 (Eq. 3) and, consequently, no chance of replication for the replicator at site *i*.

### Phenotype-genotype difference in replicability

Template-directed replication produces “enzymes” from “genes” and *vice versa* (Figure 1C and D)*.* These two forms of the same replicator species differ not only in their catalytic activities and monomer sequences. The structural feature that enables the phenotype form (“enzyme”) to be catalytically active [10] has an effect on its replicability as well. The compact 3D structure of an efficient “enzyme” form makes it difficult to copy, because it requires more energy and time to unfold during replication than in the case of loosely folded strains. That is, catalytic activity and replicability are expected to be in a trade-off relation in ribozymes [16]. The complementary “gene” form may or may not be easier to copy than the “enzyme” form, but we expect that the “gene” function would be selected for better replicability, implying that the replicability (*k*_*ip*_) of the “enzyme” form of an evolved replicator species (*i*) must be smaller than that (*k*_*ig*_) of the “gene” form of the same species (*p* and *g* in the indexes refer to “phenotype” and “genotype”, respectively). Ivica et al. [16] show that about 1.5% of short (~35 nt) random RNA sequences conform to this assumption, offering a good start for evolution to amplify the difference of phenotype to genotype replicability. The dynamical effect of this trade-off was tested with a comparison of two model versions. In Model I the replicabilities of the “enzyme” and the “gene” were the same, whereas in Model II they were different (with *k*_*ip*_ < *k*_*ig*_).

### Parasitic replicators

Parasitic replicators differ from metabolic ones in that neither of their complementary forms is enzymatically active. That is, both the “phenotype” and the “genotype” of a parasite use monomers produced by the metabolic replicator “phenotypes” for their replication, but neither form contributes to monomer production (Figure 1D).

### Replicator mobility

Replicators bind to the mineral surface reversibly, allowing them some limited mobility in the form of a slow diffusive movement on the surface. This is implemented by using the Toffoli-Margolus algorithm [18] in the model: randomly chosen *2×2* blocks of sites are rotated 90° clockwise or anticlockwise with equal (0.5) probabilities. The intensity of replicator diffusion is scaled by the average number *D* of such sub-lattice rotations per update, so that *D* = 1 means four random steps per replicator per generation on average, because one rotation moves four replicators. Note, however, that even with *D* = 0 a minimum of replicator mixing is unavoidable, because replicator movement on the surface consists of two independent components*: replicative movement (D*_*r*_*)* and *diffusive movement (D)*. The former is an inevitable consequence of the replication mechanism: putting a copy in a site adjacent to that of the template implies the movement of the copy. The relative intensity of replicative to diffusive mixing can be estimated, given that a single Toffoli-Margolus diffusion step (*D* = 1) represents 4 site swaps per replicator per generation, whereas replicative movements result in an average of 0.2 swaps per replicator in each generation (because the death rate of replicators is constant – *p*_*d*_ = 0.2 – a nd replicator densities are stationary). That is, replicative movement corresponds to *D*_*r*_ ≅ *p*_*d*_/4 = 0.05 in our model with the parameters above, assuming that the distance between the template and the empty site where the copy is placed is 1. Then the total mobility of the replicators is *D*_*t*_ = *D*_*r*_ + *D*, which yields *D*_*t*_ = 0.05 for *D* = 0. If the replication neighbourhood is larger than the von Neumann neighbourhood (the four orthogonal neighbours, Additional file 1: Figure S1) of the empty site, then the replicative component of diffusion (*D*_*r*_) is larger.

## Results

The stochastic cellular automaton consists of 90.000 sites arranged in a 300×300 rectangular square lattice. All simulations were initiated with 80% of the sites occupied at random positions by the “genotype” and “phenotype” forms of all replicator types, with both complementary forms of each replicator species represented at equal (10-10%) proportions.

Replicator coexistence has been shown to critically depend on just a small number of parameters in the original toy model of MCRS [1,6]. The three most effective determinants of coexistence were all related to *spatial mixing* in that model: the speed of replicator diffusion (*D*) and the size of replication neighbourhoods (*r*) both correspond to *replicator mobility*, whereas metabolic neighbourhood size (*h*) is the proxy for the *average distance* that metabolite (and monomer) molecules can cover on the surface before being used in a reaction, degraded or desorbed from the surface. Consequently, *h* is determined by three factors: the surface diffusibility, the degradation rate and the desorption rate of small metabolite molecules – these three components are lumped in parameter *h*.

We have screened the pheno/geno version of the model for replicator coexistence at different ranges of the same three parameters, keeping all others constant: the death rate of replicators (*p*_*d*_ = 0.2), the “claim” of the empty site (*C*_*e*_ = 2.0) and system size (*n* = 4) were the same in all simulations. Replicabilities were also fixed at *k*_*ip*_ = *k*_*ig*_ and *k*_*pp*_ = *k*_*pg*_ in Model I, and *k*_*ip*_ < *k*_*ig*_ and *k*_*pp*_ < *k*_*pg*_ in Model II (subscripts *i* and *p* denote metabolic replicators and parasite, respectively).

Each simulation produced a quasi-stationary state within the 1.000 generation time frame applied, suggesting that the underlying dynamics may admit attracting fixed point equilibria. The result of a simulation was always one of two possible outcomes: either the whole system survived (with all the metabolic replicator species present), or the replicator community died out altogether. This is just to be expected: the extinction of any one of the metabolically active species stops monomer production and thus kills all the rest, including the parasite if there is one. The survival constraint does not apply to parasites, of course: since they perform no essential function for metabolism, they may go extinct while the metabolic replicators persist.

**Figure 2:**
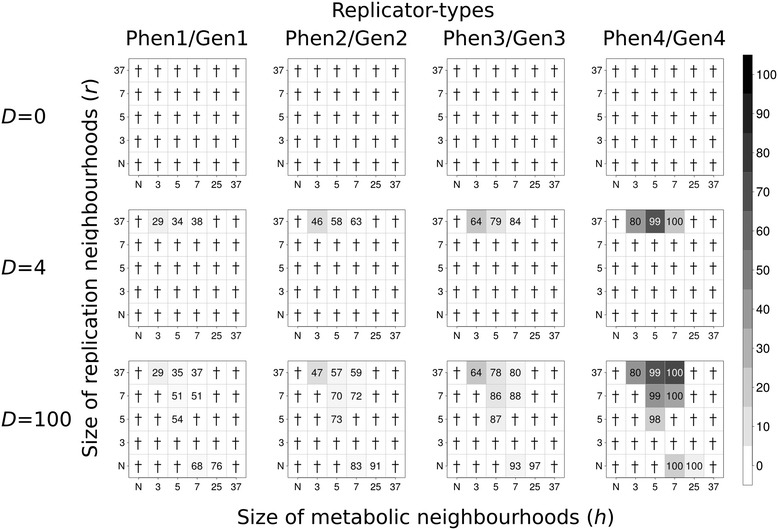
**Coexistence of metabolic replicators in Model I.** The rows of the figure differ in the number of diffusion steps per update: first row: *D* = 0, second row: *D* = 4; third row: *D* = 100. *x*- and *y*-axes are the sizes of metabolic (*h*) and replication (*r*) neighbourhood, respectively (*N*: *von Neumann* neighbourhood; 3: *3×3*, 5: *5×5*, 7: *7×7*, 25: *25×25* and 37: *37×37 Moore* neighbourhoods). The gray-scale shades of the boxes correspond to average replicator densities (%, see scale bar) on the whole grid at the end of the simulations (at *t* = 1.000). The numbers within the boxes of the panels indicate the average ratio (%) of the numbers of phenotype- and genotype forms at the end of the simulations (*100 × Phen*_*x*_ */Gen*_*x*_, *x* = 1, .., 4) in five replicate runs of the simulation in each parameter set. Zero means the collapse of the system (no replicator survives to *t* = 1.000). Replication constants: *k*_*1p*_ = 3.0, *k*_*1g*_ = 3.0, *k*_*2p*_ = 5.0, *k*_*2g*_ = 5.0 , *k*_*3p*_ = 7.0, *k*_*3g*_ = 7.0, *k*_*4p*_ = 9.0 and *k*_*4g*_ = 9.0; subscripts *p* and *g* denote phenotype-form and genotype-form of replicator types, respectively.

**Figure 3:**
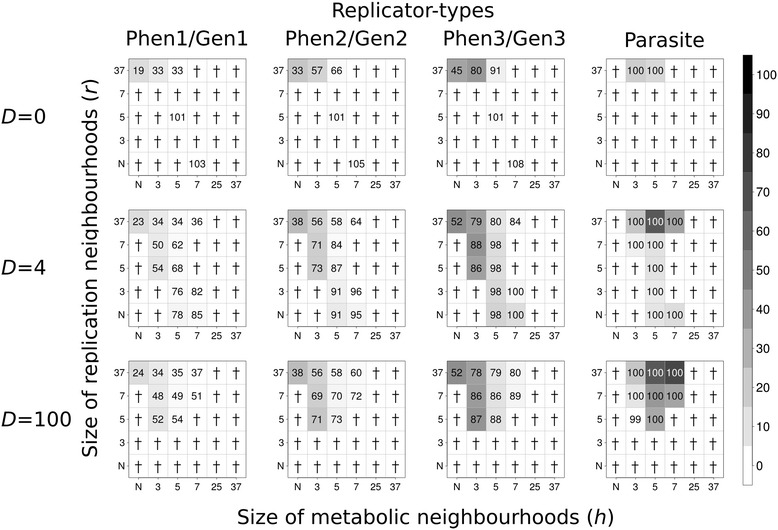
**Coexistence of metabolic and parasitic replicators in Model I.** The rows of the figure differ in the number of diffusion steps per update: first row: *D* = 0, second row: *D* = 4; third row: *D* = 100. *x*- and *y*-axes are metabolic (*h*) and replication (*r*) neighbourhood sizes respectively (*N*: *von Neumann* neighbourhood; 3: *3×3*, 5: *5×5*, 7: *7×7*, 25: *25×25* and 37: *37×37 Moore* neighbourhoods). The gray-scale shades of the boxes correspond to average replicator densities (%, see scale bar) on the whole grid at the end of the simulations (at *t* = 1.000). The numbers within the boxes of the panels indicate the average ratio of the numbers of phenotype- and genotype forms of metabolic and parasite replicators at the end of the simulations (*100 ×* Phen_x_/Gen_x_, *x* = 1, .., 3 and *Parasite* means both replicator-forms) in five replicate runs of the simulation program in each parameter set. Zeros mean that the system collapses (no replicator survives to *t* = 1.000). Replication constants of metabolic replicators: *k*_*1p*_ = 3.0, *k*_*1g*_ = 3.0, *k*_*2p*_ = 5.0, *k*_*2g*_ = 5.0 , *k*_*3p*_ = 7.0, *k*_*3g*_ = 7.0. Replication constants of parasite replicators *k*_*4p*_ = 9.0 and *k*_*4g*_ = 9.0. Subscripts *p* and *g* denote phenotype-form and genotype-form of replicator types, respectively.

**Figure 4:**
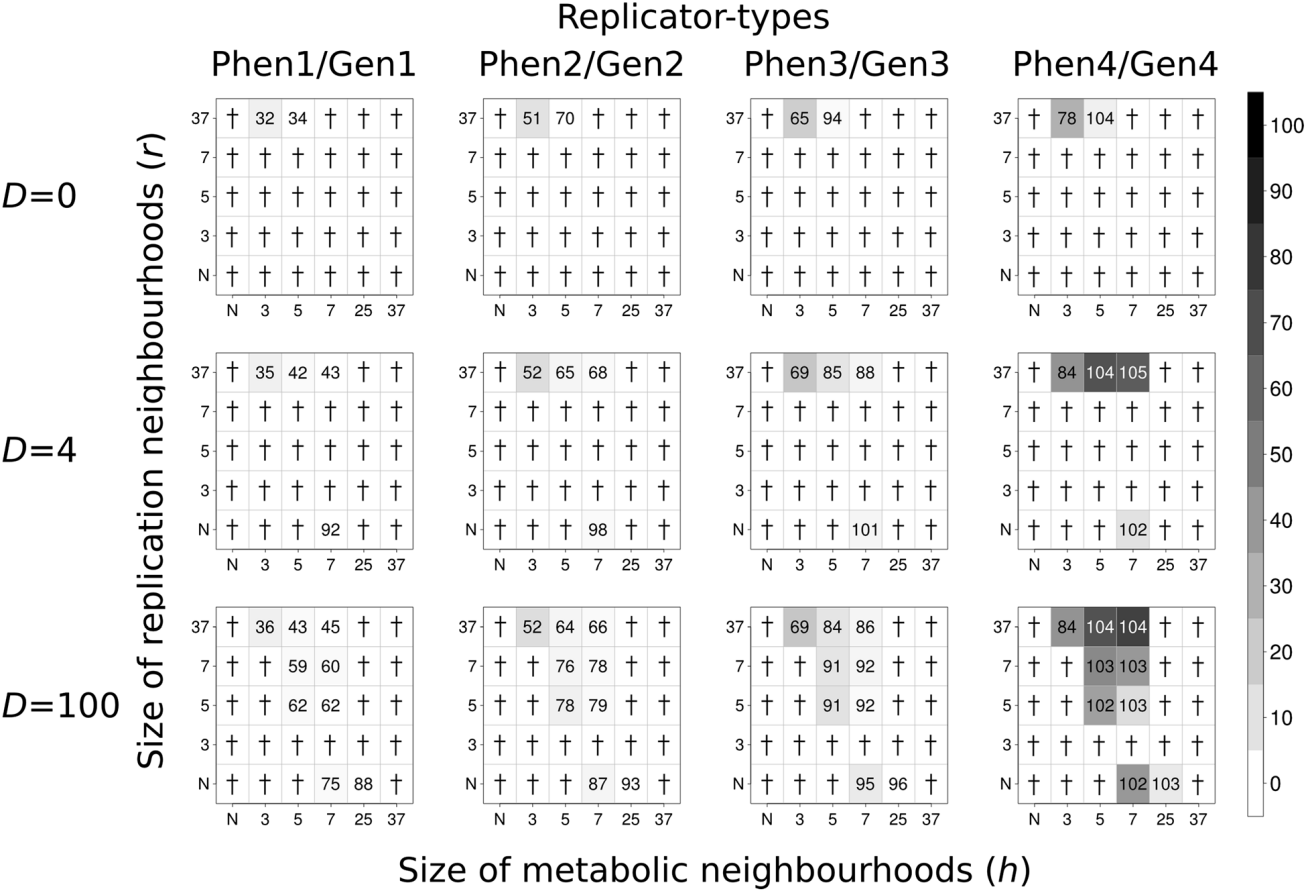
**Coexistence of metabolic replicators in Model II.** The rows of the figure differ in the number of diffusion steps per update: first row: *D* = 0, second row: *D* = 4; third row: *D* = 100. *x*- and *y*-axes are the sizes of metabolic (*h*) and replication (*r*) neighbourhood, respectively (*N*: *von Neumann* neighbourhood; 3: *3×3*, 5: *5×5*, 7: *7×7*, 25: *25×25* and 37: *37×37 Moore* neighbourhoods). The gray-scale shades of the boxes correspond to average replicator densities (%, see scale bar) on the whole grid at the end of the simulations (at *t* = 1.000). The numbers within the boxes of the panels indicate the average ratio (%) of the numbers of phenotype- and genotype forms at the end of the simulations (100 × *Phen*_*x*_ */Gen*_*x*_, *x* = 1, .., 4) in five replicate runs of the simulation in each parameter set. Zero means the collapse of the system (no replicator survives to *t* = 1.000). Replication constants: *k*_*1p*_ = 3.0, *k*_*1g*_ = 4.0, *k*_*2p*_ = 5.0, *k*_*2g*_ = 6.0 , *k*_*3p*_ = 7.0, *k*_*3g*_ = 8.0, *k*_*4p*_ = 9.0 and *k*_*4g*_ = 10.0; subscripts *p* and *g* denote phenotype-forms and genotype-forms of replicator types, respectively.

**Figure 5:**
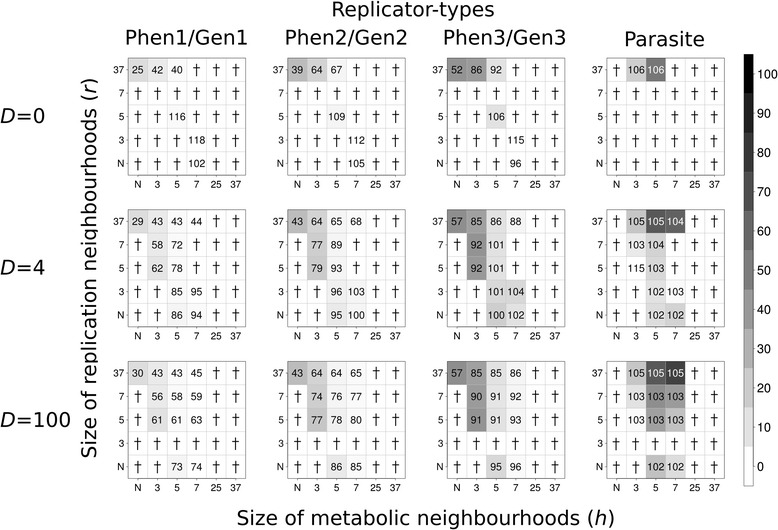
**Coexistence of metabolic and parasitic replicators in Model II.** The rows of the figure differ in the number of diffusion steps per update: first row: *D* = 0, second row: *D* = 4; third row: *D* = 100. *x*- and *y*-axes are metabolic (*h*) and replication (*r*) neighbourhood sizes respectively (*N*: *von Neumann* neighbourhood; 3: *3×3*, 5: *5×5*, 7: *7×7*, 25: *25×25* and 37: *37×37 Moore* neighbourhoods). The gray-scale shades of the boxes correspond to average replicator densities (%, see scale bar) on the whole grid at the end of the simulations (at *t* = 1.000). The numbers within the boxes of the panels indicate the average ratio (%) of the numbers of phenotype- and genotype forms of metabolic and parasite replicators at the end of the simulations (*100 ×* Phen_x_/Gen_x_, *x* = 1, .., 3 and *Parasite* means both replicator-forms) in five replicate runs of the simulation program in each parameter set. Zeros mean that the system collapses (no replicator survives to *t* = 1.000). Replication constants of metabolic replicators: *k*_*1p*_ = 3.0, *k*_*1g*_ = 4.0, *k*_*2p*_ = 5.0, *k*_*2g*_ = 6.0 , *k*_*3p*_ = 7.0, *k*_*3g*_ = 8.0. Replication constants of parasite replicators *k*_*4p*_ = 9.0 and *k*_*4g*_ = 10.0. Subscripts *p* and *g* denote phenotype-forms and genotype-forms of replicator types, respectively.

Wherever the system is persistent, its equilibrium state can be fully specified by the stationary densities of the replicator species, and the phenotype to genotype density ratio within each species. Figures 2 and 3 summarize these output data obtained with Model I to demonstrate the effect of gene/enzyme functional complementarity devoid of the replicability difference between the “enzyme” and the “gene” form of the same replicator type, without and with a parasite, respectively; Figures 4 and 5 show the same simulation outcomes for Model II. The data shown on the figures are the averages of five replicate simulations for each parameter setting.

### The effect of replicator mobility

Replicator mobility is clearly advantageous for the coexistence of the metabolic replicator community in general: increasing any one or both of the diffusion parameters (*D* and *D*_*r*_) and the size of the replication neighbourhood (*r*) has a positive effect on the persistence of the system, as well as on the average density of replicators on the surface. This conclusion is in line with our previous results [6], applies to Model I and II alike, and it is irrespective of the presence or absence of a metabolic parasite (Figures 2, 3, 4 and 5). These two parameters are almost interchangeable in terms of their direct effects: larger values of any one of them represent more mixing of replicators on the surface. This is obvious for the diffusion parameter, and easy to see for the replication neighbourhood too, because larger *r* means more distance between template and copy, so that with the replication rate left unchanged, the step length of a single diffusive move increases and thus replicative diffusion (*D*_*r*_) becomes more intensive with *r* larger.

There are a few conspicuous exceptions to the trend of monotonous increase in persistence and average replicator density with replicator mobility: at intermediate values of the replication neighbourhood size (*r*) the system goes extinct in some cases, even though at small and high *r* it is persistent (cf. Figures 2, 3, 4 and 5). We shall attempt to give a feasible explanation to this seemingly anomalous behaviour of the model in the Discussion.

### The effect of metabolite mobility

The average distance that a metabolite molecule travels on the surface before it becomes the substrate of a metabolic reaction or disappears from the system by desorption or decay is implicit in the size of the *metabolic neighbourhood* (*h*). The larger it is the longer the distance within which the products of metabolism – monomers – are available for replicators being copied. In accordance with the conclusions of all previous versions of the MCRS models we find that the pheno/geno version also admits replicator coexistence within an optimum range of metabolic neighbourhood size: very small and very large *h* values are deleterious for system persistence, for essentially the same reasons as in the previous models [1,3,5,6,17]. Very small *h* mimics the fast desorption or decay of metabolites from the mineral surface, so that most of the metabolite molecules cannot be converted to monomers before they disappear from the system. It is difficult (or, in extreme cases, even impossible) to fit a complete set of metabolic replicators into a very small metabolic neighbourhood, therefore it slows down or stops replication everywhere, for lack of monomers.

On the other hand, too large *h* reduces the advantage of rarity, because rare replicator types are available in the (large) metabolic neighbourhoods of most other replicator types which are capable of faster replication, and thus the more common types outcompete the rare type and the system collapses. In other words, increasing *h* shifts the system towards the mean-field situation in which it has been shown to go extinct invariably [1]. That is, increasing the size of the metabolic neighbourhood increases the chance of metabolic complementation while decreases the advantage of rarity. These counteracting effects are reflected in the optimal intermediate range of *h* for which coexistence occurs in the simulations (Figures 2, 3, 4 and 5).

### The effects of phenotype/genotype functional complementarity

Comparing the results of the current model (Figures 2, 3, 4 and 5) to those of a simulation study that differs from the present one only in the omission of phenotype/genotype complementarity (Additional file 2: Figure S2) shows that the persistent (*D*, *r*, *h*) parameter range of the pheno/geno version of the MCRS model is somewhat narrower than that of the non-complementary version [1,6]. This is to be expected, given that the replicator forms with the “gene” function behave somewhat like parasites in the system, using up monomers produced by the enzymatically active forms but not contributing to metabolism directly. The only difference of a “gene” from a parasite is that the copies of a “gene” are functional “enzymes”, whereas the replication of parasites yields other parasites with no metabolic functionality. This indirect functional difference is responsible for the persistence of all the “genes” (the “genome”) within the system, which does not occur with different parasites – it is only the single fastest replicating parasite that prevails in all versions of the MCRS model, all other parasites are excluded (cf. [6] Figure 3). The presence of the enzymatically inactive “gene” forms dilutes the system in terms of metabolic efficiency, which in turn naturally decreases – but does not destroy – its propensity for coexistence, especially at smaller metabolic neighbourhood sizes.

### The effects of the trade-off between gene replicability and enzyme activity

The dynamical consequences of an evolved trade-off between enzyme activity and gene replicability are clearly beneficial for the MCRS: Model I is coexistent in a section of its parameter space that is remarkably smaller than in Model II, and the average density of persistent replicator populations is also smaller everywhere in Model I (compare Figure 2 with Figure 4, and Figure 3 with Figure 5).

### The effect of parasites

The presence of parasites in the pheno/geno model makes little dynamical difference compared to the original MCRS: parasitic replicators remain persistent, sometimes at high densities, but they do not kill the system at any parameter setting studied (Figures 3 and 5). Replacing a metabolic replicator with a parasite results in a shift of the coexistent parameter range towards smaller metabolic neighbourhoods, but it is a consequence of the decrease in system size (3 metabolic replicator types instead of 4), not some kind of direct “parasite benefit”.

## Discussion

The overall dynamical behaviour exhibited by the pheno/geno version of the MCRS model is not very different from the single-sequence original [1,6]: metabolically cooperating replicators are coexistent over a large section of the physico-chemically feasible part of the parameter space, and the system is resistant to but, to some extent, also tolerant with its parasites (Figures 2, 3, 4 and 5). Coexistence is due to the general local replication advantage that rare types of metabolic replicators enjoy through a higher probability of metabolic complementation compared to more common replicator types, just like in the single-sequence model. Parasite resistance means that the parasites are not able to kill the system, due to the local metabolic disadvantage of increased parasite density: wherever parasites become abundant, metabolism breaks down and the system – including the many parasites - dies out locally, thereby decreasing parasite density. This regulatory mechanism maintains a viable balance of metabolic cooperators and parasites, the latter of which constitute a pool of replicators lacking any essential functionality in the system but always present at considerable density. Parasites are free to mutate and adopt any potentially useful function for MCRS as a whole. The possibilities include the conversion of the parasite to a new metabolic cooperator, a better replicase [3], a ribozyme that contributes to the production of membrane-constituents, or a co-factor of any of these.

### Phenotype-genotype complementarity and coexistence

Considering template-copy sequence complementarity (i.e., the phenotype-genotype distinction) does not change this picture, at least not radically. One of the obvious differences is that the coexistence regions of the parameter space, i.e., the parameter settings at which the system is viable, are shifted towards larger metabolic and replication neighbourhoods in the pheno/geno version. This is not very surprising, given that - from a functional point of view - the pheno/geno system is “diluted” by the “gene” forms. Since it is only the “enzyme” form that contributes to metabolism, and each site of the lattice can be home for a single replicator at most, the inevitable presence of the enzymatically inactive “gene” forms scales up effective neighbourhood sizes in inverse proportion to the relative frequency of “enzyme” forms.

A less obvious feature of the pheno/geno simulations is the non-monotone increase of persistence with increasing replication neighbourhood size (*r*). It shows up consistently on each of Figures 2, 3, 4 and 5. Very small replication neighbourhoods (with *r* = 4, i.e., at the von Neumann neighbourhood) seem to be advantageous for coexistence, just as large ones are. But in most cases there exists an intermediate section of the replication neighbourhood scale at which system extinction occurs. Since the persistence of the replicator system depends on the survival of the least abundant metabolic replicator species, in fact the question is: why does a very small replication neighbourhood favour the survival of the least fit (i.e., of smallest replicability) species compared to the others? The answer probably lies in the strong stochastic fluctuation of neighbourhood composition at very small neighbourhood sizes. An empty site with a single replicator in its replication neighbourhood (Additional file 1: Figure S1) has a high chance of being occupied in the next generation by a copy of that single replicator which has no competitor in the same replication neighbourhood (cf. Eqs. 1–4). If a small replication neighbourhood contains a copy of the species of smallest replicability and just a few (or even zero) copies of more common species, then it has a good chance to replicate, much better than it would had it been surrounded by many more replicators from all the fitter species. In other words, larger replication neighbourhoods may include more of the powerful competitors, thus less chance for the least fit species to reproduce. Obviously this kind of advantage of rarity can be exploited only at very small replication neighbourhoods. Larger *r* decreases this stochastic fluctuation effect, but it also increases spatial mixing due to replication movement. The former effect is deleterious, the latter is beneficial for replicator coexistence, hence the non-monotonous behaviour of system persistence with increasing replication neighbourhood.

All “enzyme” and “gene” forms are distributed on the surface homogeneously in the majority of the parameter settings, except at large metabolic *and* very small replication neighbourhoods (towards the bottom-right region of the panels in Figures 2, 3, 4 and 5), where the spatial pattern of the system appears patchy with the patches randomly moving across the lattice (see Additional file 3: Movie S1 and Additional file 4: Movie S2). This patchiness, just as the non-monotone behaviour of the system with increasing replication neighbourhood, is the result of another delicate balance of two antagonistic effects. At very small *r* the system is sparse, so that survival is possible only at locations where the least fit replicator species is present. Each rare type replicator has a “court” of more common species around it, and the patch thus produced is persistent, provided that the system is not mixed too much to drift cooperating replicators far apart (i.e., at small *D*) and that the metabolic neighbourhood *h* is sufficiently large to include the whole patch. Then all the replicators of a patch fit into the same metabolic neighbourhood so that they are all supplied by monomers for copying. Very small *r* guarantees that the rare type survives in spite of *h* being quite large. Of course, too large metabolic neighbourhoods are fatal for the system because of the shift towards mean-field dynamics leading to system extinction, so the viable range of *h* at very small *r* is limited. Note that the sharp borders of the patches dissolve with increasing diffusion, and the pattern of the arena becomes “normal”, i.e., nearly homogeneous.

### Shifts in “enzyme/phenotype” to “gene/genotype” strain proportions

The replication of the “gene” form of a certain species yields the “enzyme” form of the same species, but only in the presence of all the metabolically active “enzyme” forms within the metabolic neighbourhood of the strain to be copied. The functional asymmetry of “gene” and “enzyme” forms is reflected in the asymmetry of their copy numbers: the “enzyme” form is always underrepresented in the simulated replicator populations (cf. the proportions given as numbers on Figures 2, 3, 4 and 5). This shift in “enzyme” to “gene” proportions is due to the fact that “enzymes” are always more likely to be copied than “genes”, because they are active members of their own metabolic neighbourhoods. Takeuchi and his co-workers come to a similar conclusion with their cellular automata model of an evolving gene-enzyme pair: the gene strand tends to dominate the population [19]. The effect is analogous to the advantage of rarity – we may call it the “*advantage of phenotypes*” - : since “enzymes” replicate more often and they always yield “genes”, it is the latter that become more abundant. This is especially conspicuous in the case of Model I (Figures 2 and 3), where the replicabilities of the “enzyme” and the “gene” forms are the same for each species. We will see in the next section that the consequential loss in system persistence may be compensated by assuming a straightforward trade-off between enzymatic activity and replicability, which in turn may be the result of prebiotic selection for a better “division of labour” between phenotype and genotype strains.

### Enzyme activity/replicability trade-off

Model I differs from Model II in that the latter assumes smaller replicability for the “enzyme” form of a replicator species than for the “gene” form of the same species. The resulting trade-off between enzyme activity and replicability has a few consistent dynamical effects in MCRS: 1) the system is persistent in a considerably larger part of the parameter space; 2) wherever the system persists in both models, Model II produces larger overall densities and 3) higher relative frequencies of the phenotype (“enzyme”) form (by compensating the *advantage of phenotypes,* compare Figure 2 to Figure 4 and Figure 3 to Figure 5).

Recall that the enzyme activity part of the trade-off is a physico-chemical necessity: an efficient ribozyme needs to be relatively compact and energetically stable, in order to present the required spatial configuration of critical residues in a steady structure for executing the reaction it catalyses. The replicability part of the trade-off (*k*_*p*_ < *k*_*g*_) is not so straightforward, because nothing guarantees *ab ovo* that the complementary strain of a good ribozyme has a loose spatial structure, which would make it easier to copy. To the contrary – the complementary strains of efficient ribozymes have a high chance of being compact themselves too - but this is not a strictly constrained relation either [16]. Therefore, the phenotype/genotype trade-off, the “division of labour” proposed by Ivica et al. [16] between complementary strains of the same replicator species is a possible, but not inevitable feature of the “enzyme”/”gene” pair [20]. The fact that this trade-off is beneficial for the metabolic replicator system as a whole makes it very probable that it will be selected for, so that *k*_*p*_ < *k*_*g*_ would eventually appear as an evolved feature of the replicator species. This is a conjecture to be studied in detail later, using the MCRS model framework combined with the Vienna algorithm of RNA folding [21]. Regardless of what mechanism the “enzyme”/”gene” trade-off is achieved by, it can be regarded as the first, prebiotic evolutionary step towards a simple form of phenotype amplification (the production of many enzymes from a single gene) – a feature of all recent organisms in which it is attained through transcriptional and translational amplification.

## Conclusion

Incorporating complementary template replication and the consequent phenotype-genotype differentiation into the MCRS framework does not change the basic dynamical properties of the system: all previous conclusions about replicator coexistence and resistance against parasites [1,6] still hold. We do not know – therefore it calls for further studies – whether other theoretical approaches to prebiotic evolution (e.g. SCM – [22], Hypercycle – [23]) are also robust against the same modification?

The functional asymmetry of complementary RNA strains is the cause of the shift in phenotype/genotype proportions in MCRS, to the advantage of genotypes. We see the opposite asymmetry in recent organisms: phenotypes (enzymes) are amplified to many copies from just a few copies of their genes. The first step of phenotype amplification might have been the evolutionary acquisition of the enzymatic activity/replicability trade-off which we have shown to be advantageous for the coexistence and the dynamical efficiency of MCRS. More advanced ways of functional differentiation or “division of labour” (including the adoption of DNA and the complicated machineries of transcription and translation) might have developed later in some vesicular forms of the metabolic replicator system (see [20]) on the evolutionary route towards the recent DNA-RNA-protein World.

## Availability of supporting data

Additional file 1: Figure S1. introduces the types of neighbourhoods and Additional file 2: Figure S2 shows the results of the original model lacking template complementarity (from [6] Figure 2) Movies show some representative time courses of Model II with the development of heterogeneous replicator patches at different diffusion intensities which either support (Additional file 3: Movie S1 – *D* = 4) or hamper (Additional file 4: Movie S2 – *D* = 0) the persistence of parasites.

## Additional files

Additional file 1: Figure S1. Neighbourhoods used in the model. The light grey rectangles depict the Moore (*Panel* ***A*** *)* and the von Neumann (*Panel* ***B*** *)* types of neighbourhoods around *X,* which may be a replicator or an empty site depending on the function of the neighbourhood (metabolic or replication neighbourhood). Additional file 2: Figure S2. Coexistence of metabolic replicators as the function of replicator diffusion (*D*), metabolic (*h*) and replication (*r*) neighbourhood size. The panels of the figure differ in the number of diffusion steps per generation: Panel **A:** *D* = 0, Panel **B:** *D* = 1, Panel **C:** *D* = 4 and Panel **D:** *D* = 100. x- and y-axes are the sizes of metabolic neighbourhoods (*h*) and replication neighbourhoods (*r*) respectively (N: von Neumann neighbourhood; 3: 3 × 3, 5: 5 × 5, 7: 7 × 7, 25: 25 × 25 and 37: 37 × 37 Moore neighbourhoods). The grayscale shades correspond to average replicator densities (%) on the whole grid at the end of the simulations (i.e., for t = 1.000). The numbers within panels indicate coexistent/extinct replicate simulations out of the five repetitions with the same parameter set and different pseudo-random number sequences. From [6]. Additional file 3: Movie S1. Heterogeneous patches on the grid at *D* = 4. The spatial dynamics of replicator-types on the lattice from generation 1 to 1000 in Model II. Color shade codes: *Light colors*: the phenotype (“enzyme”) forms; *Dark colors:* the genotype (“gene”) forms of the same replicator type. *Green:* replicator type 1; *Blue*: replicator type 2; *Red*: replicator type 3; *Yellow/orange*: parasitic replicator type. Other parameters: the size of metabolic neighbourhoods is *h* = *5x5 (Moore-neighbourhood)*, the size of replication neighbourhoods is *r* = 4 (*von Neumann* neighbourhood). Replication constants: *k*_*1p*_ = 3.0, *k*_*1g*_ = 4.0, *k*_*2p*_ = 5.0, *k*_*2g*_ = 6.0, *k*_*3p*_ = 7.0, *k*_*3g*_ = 8.0, *k*_*4p*_ = 9.0 and *k*_*4g*_ = 10.0; subscripts *p* and *g* denote phenotype and genotype forms of replicator types, respectively. Additional file 4: Movie S2. Heterogeneous patches on the grid at *D* = 0. Color codes and parameters as in Additional file 3: Movie S1, except for the replication neighbourhood, which is 5x5 (*Moore*).
