# Supplementary figures and images for: Phenotype/genotype sequence complementarity and prebiotic replicator coexistence in the metabolically coupled replicator system
Source: BMC Evol Biol. 2014 Nov 25;14:234. doi: 10.1186/s12862-014-0234-8 (PMC4256930; doi:10.1186/s12862-014-0234-8)

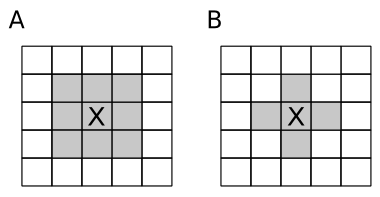

Supplement: Additional file 1: Figure S1. — Neighbourhoods used in the model. The light grey rectangles depict the Moore (Panel A ) and the von Neumann (Panel B ) types of neighbourhoods around X, which may be a replicator or an empty site depending on the function of the neighbourhood (metabolic or replication neighbourhood). [file 12862_2014_234_MOESM1_ESM.png]

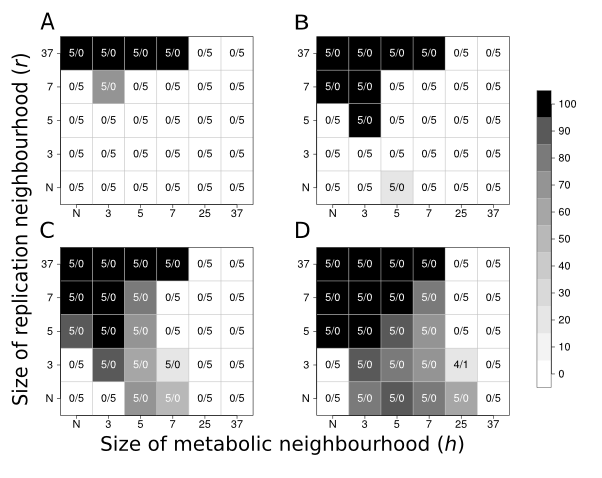

Supplement: Additional file 2: Figure S2. — Coexistence of metabolic replicators as the function of replicator diffusion (D), metabolic (h) and replication (r) neighbourhood size. The panels of the figure differ in the number of diffusion steps per generation: Panel A: D = 0, Panel B: D = 1, Panel C: D = 4 and Panel D: D = 100. x- and y-axes are the sizes of metabolic neighbourhoods (h) and replication neighbourhoods (r) respectively (N: von Neumann neighbourhood; 3: 3 × 3, 5: 5 × 5, 7: 7 × 7, 25: 25 × 25 and 37: 37 × 37 Moore neighbourhoods). The grayscale shades correspond to average replicator densities (%) on the whole grid at the end of the simulations (i.e., for t = 1.000). The numbers within panels indicate coexistent/extinct replicate simulations out of the five repetitions with the same parameter set and different pseudo-random number sequences. From [6]. [file 12862_2014_234_MOESM2_ESM.png]
